# High-Salt Diet Induces Depletion of Lactic Acid-Producing Bacteria in Murine Gut

**DOI:** 10.3390/nu14061171

**Published:** 2022-03-10

**Authors:** Ibrahim Hamad, Alessio Cardilli, Beatriz F. Côrte-Real, Aleksandra Dyczko, Jaco Vangronsveld, Markus Kleinewietfeld

**Affiliations:** 1 VIB Laboratory of Translational Immunomodulation, Center for Inflammation Research (IRC), Hasselt University, 3590 Diepenbeek, Belgium; ibrahim.hamad@uhasselt.be (I.H.); alessio.cardilli@uhasselt.be (A.C.); beatriz.cortereal@uhasselt.vib.be (B.F.C.-R.); aleksandra.dyczko@uhasselt.be (A.D.); 2 Department of Immunology and Infection, Biomedical Research Institute (BIOMED), Hasselt University, 3590 Diepenbeek, Belgium; 3 Research Group for Environmental Biology, Centre for Environmental Sciences, Hasselt University, 3590 Diepenbeek, Belgium; jaco.vangronsveld@uhasselt.be

**Keywords:** gut microbiome, sodium chloride (dietary), dysbiosis, bacterial metabolites, inflammation

## Abstract

Dietary habits are amongst the main factors that influence the gut microbiome. Accumulating evidence points to the impact of a high-salt diet (HSD) on the composition and function of the intestinal microbiota, immune system and disease. In the present study, we thus investigated the effects of different NaCl content in the food (0.03%/sodium deficient, 0.5%/control, 4% and 10% NaCl) on the gut microbiome composition in mice. The bacterial composition was profiled using the 16S ribosomal RNA (rRNA) gene amplicon sequencing. Our results revealed that HSD led to distinct gut microbiome compositions compared to sodium-deficient or control diets. We also observed significant reduction in relative abundances of bacteria associated with immuno-competent short-chain fatty acid (SCFA) production (*Bifidobacterium*, *Faecalibaculum*, *Blautia* and *Lactobacillus*) in HSD-fed mice along with significant enrichment of *Clostridia*, *Alistipes* and *Akkermansia* depending on the sodium content in food. Furthermore, the predictive functional profiling of microbial communities indicated that the gut microbiota found in each category presents differences in metabolic pathways related to carbohydrate, lipid and amino acid metabolism. The presented data show that HSD cause disturbances in the ecological balance of the gastrointestinal microflora primarily through depletion of lactic acid-producing bacteria in a dose-dependent manner. These findings may have important implications for salt-sensitive inflammatory diseases.

## 1. Introduction

The human gastrointestinal (GI) tract harbors a complex community of microorganisms, which include bacteria, fungi, viruses and protists, nominated the gut microbiota (GM). The GM exerts effects on the host during homeostasis and disease [1]. In recent years, the human GM has emerged as an area of utmost interest, considering the impact that the microbiota have on health and disease [2]. The reduction in gut bacterial diversity (dysbiosis) has been linked to the pathogenesis of many inflammatory and infectious diseases [1,2,3].

A complex dynamic network regulates the relationship between the host and the gut microbiome [4]. This relationship is constantly challenged by both genetic and environmental factors and can lead to significant alterations of the microbial community structure [1]. Diet is regarded as one of the main players in altering the GM across the life span [1,3,5]. Intestinal health-promoting bacteria play an important role in keeping immune and metabolic homeostasis by short-chain fatty acid (SCFA) production, and it is possible to influence bacterial SCFA production through dietary approaches [6,7]. Many gut bacteria-associated diseases have intensely increased over the past century, suggesting that a change in lifestyle might alter the gut microbiota symbiosis with the host due to the depletion of symbiotic and protective microbes [8]. In fact, Western-style diet, characterized by high levels of saturated fat and sugars, may irreversibly deplete microbial diversity and lead to the loss of specific beneficial bacterial taxa in the gut [9]. Accordingly, the low consumption of dietary fibers may play a role in eradicating specific microbial taxa [10]. 

Western diet is further distinguished by high salt content. The high salt consumption is a recognized risk factor for obesity, hypertension and cardiovascular disease and has been associated with autoimmunity [11,12,13]. High-salt diet (HSD) has been proven to have diverse effects on GI functions [14,15], and Western countries characterized by HSD consumption show a higher prevalence of autoimmunity, such as inflammatory bowel disease (IBD) [16,17,18]. In mice, HSD was shown to be correlated to inflammatory response from small intestine and colonic mucosal immunity, leading to exacerbation of the DSS-induced colitis [19]. HSD-promoted inflammatory effects could be explained by an increased induction or activation of pro-inflammatory immune cells like T helper 17 cells (Th17) [20,21], IL-17-producing innate lymphoid type 3 cells (ILC3) [17], dendritic cells (DCs) [22] and M1-like macrophages [23], and a parallel inhibition of immune suppressive cell types like regulatory T cells (Tregs) or M2-like macrophages [24,25]. 

Previous work from Wilck et al. [26] has shown a significant depletion of *Lactobacillus* spp. after HSD and further demonstrated the beneficial effect of *Lactobacillus murinus* on salt-sensitive hypertension and experimental autoimmune encephalomyelitis (EAE). Reduction of dietary sodium increases circulating SCFAs, supporting the idea that dietary sodium may have an impact on the gut microbiome in humans [27]. Moreover, it was previously shown that increases in SCFAs are linked with an improvement in both blood pressure and arterial function [28]. Thus, the potential common effect of HSD on GM by decreasing the relative abundance of *Lactobacillus* [19,26], possibly correlates to a decrease of microbial SCFA production [19,27,29]. 

So far, few studies have examined the shifts on the gut microbiome composition in response to HSD. A study by Miranda et al. [19], demonstrated that HSD-derived gut microbiota was crucial for HSD-induced colitis. Further, they observed a reduced production of the SCFA butyrate [19]. Moreover, two other independent studies investigating the impact of HSD on gut microbiota uncovered a link between HSD and the promotion of gut leakage and renal injury [30], as well as protein digestion by modulating proteins secreted by the gut microbiota [14]. 

A recent study attempted to evaluate the potential shared effects that HSD could imprint on both human and mice GM signature. In this study, both GM were characterized by an increase of the phyla Firmicutes and Proteobacteria as well as the genus *Prevotella* [22]. Interestingly, HSD led to a significant depletion of potential lactic acid-producing bacteria belonging to family Leuconostocaceae in the murine gut ecosystem. On the other hand, the group detected a significant loss on the relative abundance of Bacillales, *Weissella* and Streptococcaceae [22].

Despite the current knowledge on the impact of HSD on the gut microbiome, all previous studies have mainly focused only on one range of HSD, without considering any possible quantitative effect on the gut microbial communities. In our study, we evaluated the effect of different ranges of HSD and their impact on the microbial variation. Furthermore, we investigated whether this effect is accompanied by an alteration in microbial functional signatures or taxonomic shifts in response to diverse concentrations of NaCl in the diet in a dose-dependent manner.

## 2. Method and Materials 

### 2.1. Animals and Diet

Male wild-type C57BL/6 mice (7–8 weeks old) were purchased from Charles River and housed in the facility of the University of Hasselt under standardized conditions. Animal studies were approved by the Ethical Committee on Animal Experiments (ECAE) Hasselt University (ID201618A4V1). Mice were group housed (4 mice/cage) in ventilated cages in a temperature-controlled room (21–23 °C) with a 12:12 h light/dark light cycle. The following purified diets were purchased from Ssniff (Soest, Germany): 0.03% NaCl/sodium deficient (E15430-24), 0.5% NaCl/control diet (E15430-04), 4% NaCl/HSD (E15431-34) and 10% NaCl/HSD (E15432-44). Drinking water for HSD animals (4% and 10% NaCl) was supplemented with 1% NaCl.

### 2.2. DNA Extraction

For amplicon-based 16S rRNA gene sequencing, fecal pellets were collected and kept at −80 °C until processing. DNA was extracted using an established protocol [31]. Briefly, murine pellets were placed in a 2-mL tube containing a 200-mg mixture of 0.1–0.5 mm glass beads and 1.5 mL of lysis buffer (ASL) (Qiagen, Hilden, Germany). Mechanical disruption was made by bead-beating the mixture. A minor modification was made to the manufacturer’s procedure by prolonging the proteinase K incubation time to 2 h at 70 °C. For all DNA extractions, 200 µL of distilled water was used as a negative control. The extracted DNA was stored at −20 °C until use.

### 2.3. 16S rRNA Gene Amplification and Sequencing

The V4 region (F515/R806) of the 16S rRNA gene was amplified according to previously described protocols [32]. Briefly, for DNA-based amplicon sequencing, 25 ng of DNA was used per PCR reaction (30 μL). The PCR conditions consisted of initial denaturation for 30 s at 98 °C, followed by 25 cycles (10 s at 98 °C, 20 s at 55 °C, and 20 s at 72 °C). Each sample was amplified in triplicates and subsequently pooled. After normalization, PCR amplicons were sequenced on an Illumina MiSeq platform PE300 (Illumina, Inc., San Diego, CA, USA).

### 2.4. Analysis and Processing of 16S rRNA Gene Sequencing Data

Raw sequences were processed using a QIIME2 [33] pipeline. After length and quality filtering (default parameters), reads were filtered and assigned into operational taxonomic units (OTUs) using DADA2 [34]. Taxonomic assignment was performed by the VSEARCH algorithm [35] and the Silva database v128. Chimeras were excluded during the analysis. Alpha-diversity was assessed using two different metrics: richness and Faith’s phylogenetic diversity. Weighted and unweighted UniFrac distances [36] were used to create principal coordinates analysis (PCoA) graphs. In order to estimate the alpha diversity, the whole OTU count table was rarefied to 40,000 sequences per sample 100 times. All statistical analyses were conducted using the R software (https://www.R-project.org/; accessed on 1 January 2022; Version 4.1.0). PCoA was generated using the “vegan” package (Version 2.5-7) [37]. Packages and data separation were tested by permutation test with pseudo-F ratios (function “Adonis” in “vegan”). Conditional correspondence analysis (CCA) was also performed to determine the relationship between the gut microbiota composition and different NaCl content in the food. Analysis of similarity was performed by anosim function from “vegan” (version 2.5-6) package of R: *p* value = 0.001. To compare the changes in bacterial composition among groups, a preliminary analysis with Wilcoxon rank-sum test or Kruskal–Wallis test was performed. When necessary, *p* values were corrected for multiple comparisons using the Benjamini–Hochberg method. A false discovery rate (FDR) ≤ 0.05 was considered as statistically significant. The functional differences between microbiomes of different NaCl content in the food (0.03%, 0.5%, 4% and 10% NaCl) were analyzed by PICRUSt, a bioinformatics software package to predict metagenome functional content from 16s rRNA gene sequencing data (http://picrust.github.io/picrust/; accessed on 1 January 2022; PICRUSt 1.1.4). PICRUSt2 (Version 2.4.1)was also used for the predictions of functional metabolic pathways [38]. Briefly, the raw count data was used as an input file for the Python programming environment and run through the PICRUSt2 pipeline with default parameters, obtaining a matrix with samples as columns and individual bacterial metabolic pathways as rows. The matrix with the predictive function abundances was then processed and normalized by log10 transformation and Pareto scaling. Finally, the differences between groups were statistically compared in R software using Wilcoxon test and Kruskal-Wallis test functions and *p* values adjusted by the Holm method.

## 3. Results

### 3.1. Gut Microbiota Diversity in Dependence to Different Levels of Sodium Intake

**Figure 1:**
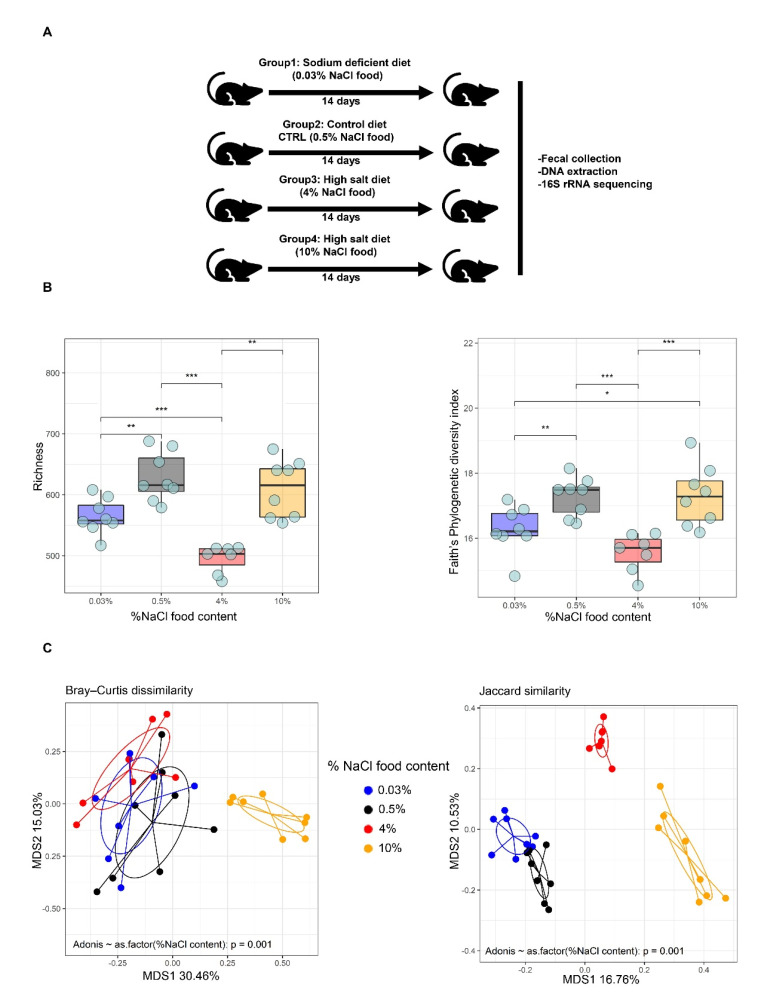
Impact of salt on the composition and structure of the murine gut microbiome. (**A**) Experimental design. C57BL/6 male mice were fed on sodium-deficient 0.03% NaCl, 0.5% NaCl (control), high salt 4% NaCl (HSD) and high salt 10% NaCl (HSD), then the bacterial composition of the mouse gut was profiled using the 16S rRNA amplicon sequencing. (**B**) Alpha diversity analysis of fecal microbiota, richness (left) and Faith’s phylogenetic diversity (PD) (right). (**C**) Principal coordinate analysis plot of beta diversity measure Bray-Curtis dissimilarity (left), PCoA based on Jaccard’s similarity calculated from the presence/absence of taxa represented on the 16S rRNA gene (right). * *p* ≤ 0.05—; ** *p* ≤ 0.01—; *** *p* ≤ 0.001.

To determine the impact of different levels of salt intake on the composition and structure of the murine gut microbiome, fecal pellets from C57BL/6 mice fed with diets differing only in their sodium chloride content (0.03%, 0.5%, 4% and 10% NaCl) were collected and subjected to 16S rRNA gene amplicon sequencing (Figure 1A). A total of 2,160,688 reads from the V4 region of the 16S rRNA gene were obtained using the MiSeq sequencing system. Among these, 67.214 ± 26.086 (mean ± SD) high-quality reads per sample were selected for analysis. The rarefaction curves of most samples reached a plateau at a minimum cut-off of 97% (Figure S1). To better understand the ecological impact of HSD on bacterial communities, OTU tables were used to generate alpha (richness, Faith’s Phylogenetic Diversity and Shannon) and beta diversity (Bray-Curtis dissimilarity, Jaccard similarity, weighted and unweighted UniFrac) indexes (Figure 1B,C, Figure S2–S4). The microbial richness was calculated in the fecal samples collected from the mice of the different groups (0.03%, 0.5%, 4% and 10% NaCl). Both taxonomic/non-phylogenetic and phylogenetic alpha diversity indexes (Faith’s phylogenetic diversity) were estimated, and the results revealed a significant difference in alpha diversity index between the groups (Figure 1B). Interestingly, the richness and Faith’s phylogenetic diversity index were significantly lower in mice fed with 4% NaCl compared to the 0.03% and 0.5% NaCl groups (Figure 1B). Previous studies have also detected a tendency in reduction in the alpha diversity index when comparing HSD to controls [26,39]. However, other studies did not observe changes in the alpha diversity index after HSD [14,19]. Furthermore, our results showed a significantly higher richness in mice fed with 10% NaCl compared to 4% NaCl. The differences in the gut microbiome diversity between the 4% and 10% HSD are surprising and might point to partial changes in the gut environment that leads to a depletion of some bacterial taxa favoring the expansion of other halotolerant bacteria, such as members of Staphylococcaceae. Further, despite not detecting differences in the overall bacterial load between the 0.5% NaCl and 10% NaCl groups, the bacterial OTUs found in each group were relatively distinct (Figures S2 and S4). This observation is contradictory to the relative decrease in species biodiversity previously detected by Ferguson et al. [22], although the group did not reach a statistically significant effect and mice were fed with 8% NaCl HSD instead of 10% NaCl.

Similar to 4% NaCl-fed mice, the community diversity decreased in the 0.03% NaCl group compared to the control group (0.5% NaCl). Although analyses using richness and Faith’s Phylogenetic diversity index indicated differences between bacterial communities among groups, these differences were not confirmed by the Shannon diversity index (Figure S3).

To compare the overall compositions of fecal microbiota among all samples, a PCoA was performed. Distances between centroids of each group were tested by permutation test with pseudo-F ratios. Both taxonomic/non-phylogenetic indexes (Bray-Curtis dissimilarity and Jaccard similarity) and phylogenetic indexes (weighted and unweighted UniFrac) were estimated. Based on the beta diversity metrics, PCoA showed a clear separation of the gut microbiota composition between 0.03%, 0.5%, 4% and 10% NaCl groups. The microbial-based community analysis revealed that the gut microbiota structure varied significantly between HSD groups (4% and 10% NaCl) (Figure 1C and Figure S4). Furthermore, a slight change was observed in the unweighted UniFrac and Jaccard similarity metrics between the control group and the 0.03% NaCl group (Figure 1C and Figure S4). While the 10% NaCl fed mice group clustered separately in every metric, the 4% NaCl group only displayed different clustering with the remaining groups for the unweighted UniFrac and Jaccard similarity metrics (Figure 1C and Figure S4). These results can be explained considering how each metric is calculated. While unweighted UniFrac and Jaccard similarity metrics are based only on the presence/absence of taxa, weighted UniFrac and Bray-Curtis dissimilarity are normalized on the relative abundance of each taxon. So, while weighted UniFrac and Bray-Curtis dissimilarity take into account the core-microbiota differences, the unweighted UniFrac and Jaccard similarity metrics consider only the qualitative difference between groups (since rare taxa will be weighted like the more abundant taxa). Therefore, in the 10% NaCl group, the compositional shifts were linked with both the core microbiota species as well as more rare bacteria. On the other hand, the independent cluster of the 4% NaCl group indicates that these mice were characterized from microbial shifts that involved mainly rare bacteria. 

Our results reveal that high salt intake leads to alterations in the microbiome alpha and beta diversity. These changes are evident by distinct clustering between the different groups, demonstrating that microbiota structure is altered in dependence of different sodium intake levels.

### 3.2. HSD Depletes Lactic Acid-Producing Bacteria

**Figure 2:**
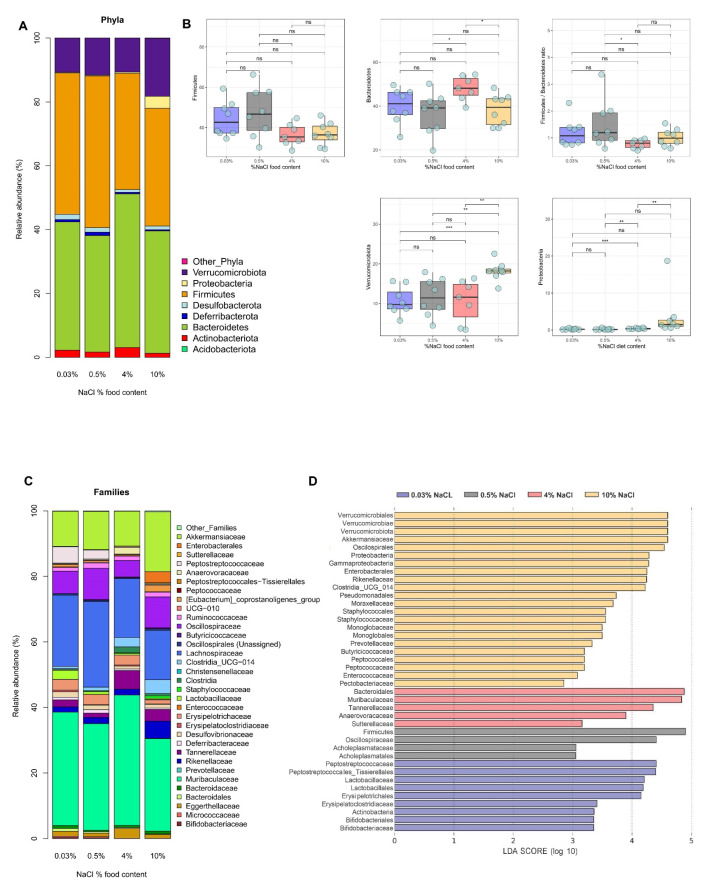
Alteration of microbiota induced by the HSD. Microbial taxonomic profiles from the fecal contents of mice fed on sodium-deficient 0.03% NaCl, 0.5% NaCl (control), high salt 4% NaCl (HSD) and high salt 10% NaCl (HSD). (**A**) Fecal microbial profile at the phylum level. The X-axis represents the group name, and the Y-axis represents the relative abundance of each taxon. (**B**) Bacterial phyla differentially represented in feces of mice fed on sodium-deficient 0.03% NaCl, 0.5% NaCl (control), high salt 4% NaCl (HSD) and high salt 10% NaCl (HSD). (**C**) Fecal microbial profile at the family level. The X-axis is the sample name or group name, and the Y-axis is the relative abundance (taxon reads/total reads in the gut microbiota). (**D**) Bacterial families differentially represented in feces of mice fed on sodium-deficient 0.03% NaCl, control 0.5% NaCl, high salt 4% NaCl (HSD) and high salt 10% NaCl (HSD) (left). Linear discriminative analysis (LDA) effect size (LEfSe) analysis of the gut microbiota among mice fed on sodium deficient 0.03% NaCl, control 0.5% NaCl, high salt 4% NaCl (HSD) and high salt 10% NaCl (HSD) (right). * *p* ≤ 0.05; ** *p* ≤ 0.01; *** *p* ≤ 0.001.

To evaluate the overall composition of intestinal bacterial communities in fecal samples of mice fed with different NaCl content in their diet, we analyzed the similarity of bacterial taxonomy at the phylum, family and genus levels. At the phylum level, the most abundant taxa obtained were Firmicutes (47.5% mean ± 18.5% SD), Bacteroidetes (37% mean ± 17.3% SD), Verrucomicrobiota (13% mean ± 9.5% SD), Proteobacteria (9.3% mean ± 9.36% SD) and Actinobacteriota (4.85% mean ± 4.85% SD) (Figure 2A). Interestingly, both Proteobacteria and Verrucomicrobiota relative abundances showed a significant enrichment in 10% NaCl compared to all the other groups (Figure 2B). 

Additionally, we observed a trend that both 4% and 10% HSD led to a depletion of bacteria of phylum Firmicutes and further enrichment of the phylum Bacteroidetes. Accordingly, we observed a decreased tendency in the Firmicutes/Bacteroidetes ratio following both 4% and 10% NaCl HSD (Figure 2B). Our data contradict with the findings from Ferguson et al. [22]; in their study, a significant Firmicutes enrichment was detected as well as a depletion in Bacteroidetes after HSD. 

At the family level, the highest microbial shift was observed in 4% and 10% NaCl compared to the sodium-deficient (0.03% NaCl) and control (0.5% NaCl) groups (Figure 2C,D and Figure S5). Bifidobacteriaceae, Peptostreptococcaceae and Lactobacillaceae were depleted in a dose-dependent manner from 0.03% NaCl to 10% NaCl fed mice (Figure 2D and Figure S5). The observed Lactobacillaceae depletion after HS exposure was consistent with several previous studies examining the impact of HSD on the gut microbiome [14,26,39]. However, to our knowledge, no other study has identified a reduction of the Bifidobacteriaceae and Peptostreptococcaceae family after HSD. On the other hand, bacteria belonging to the Akkermansiaceae, Rikenellaceae and Staphylococcaceae families were only enriched in 10% NaCl compared to all other groups (Figure 2D and Figure S5). Interestingly, members of the Prevotellaceae family were also shown to be overrepresented in the 10% NaCl group (Figure 2D), and previous studies have linked bacterial taxa belonging to Prevotellaceae with chronic inflammation, including rheumatoid arthritis [40,41].

**Figure 3:**
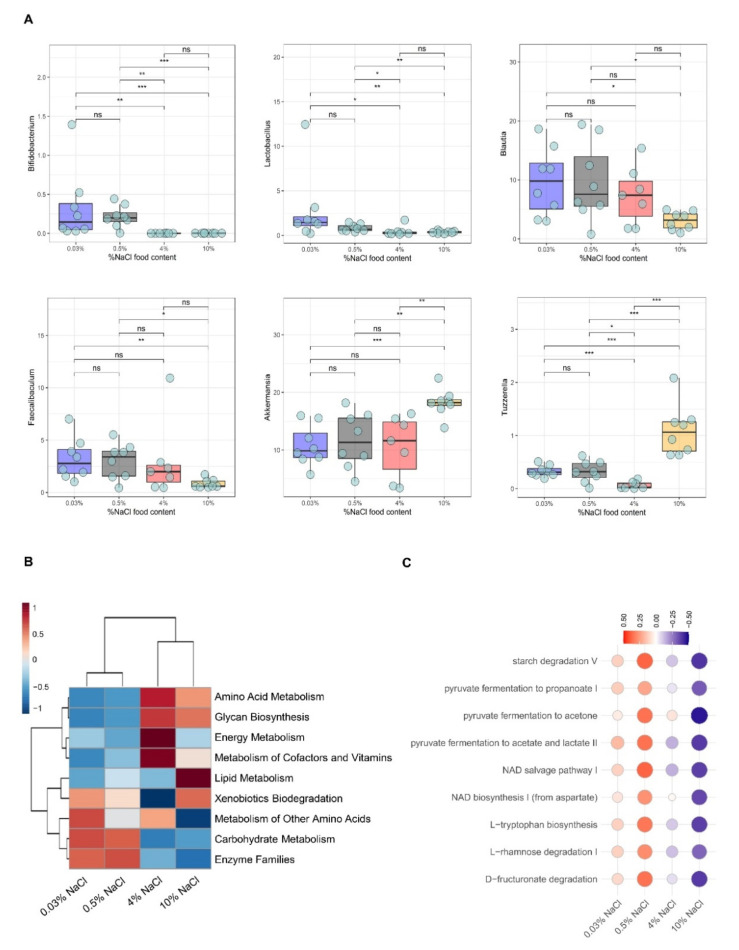
HSD associated with depletion of lactic acid-producing bacteria. (**A**) Bacterial genera differentially represented in feces of mice fed on sodium-deficient 0.03% NaCl, 0.5% NaCl (control), high salt 4% NaCl (HSD) and high salt 10% NaCl (HSD). (**B**) Predicted functional analysis of the gut microbiota using the PICRUSt software tool. Heat map shows the relative abundance changes of functional profile of gut microbiota among mice fed on sodium-deficient 0.03% NaCl, 0.5% NaCl (control), high salt 4% NaCl (HSD) and high salt 10% NaCl (HSD). (**C**) The heatmap representing predicted function abundances per OTUs computed by PICRUST2 pipeline. The relative change of values indicated by color: red color shows an enrichment of the predictive function abundances, and blue indicates a depletion of the predictive function abundances (0.5% NaCl group was used as a control). * *p* ≤ 0.05; ** *p* ≤ 0.01; *** *p* ≤ 0.001.

We further analyzed the taxonomic similarity of bacteria at the genus level. Our results showed that lactic acid-producing bacteria, such as *Bifidobacterium* and *Lactobacillus* were gradually depleted from 0.03% to 10% NaCl-fed mice groups (Figure 3A). Additionally, the relative abundance of other lactic acid-producing bacteria, such as *Blautia* and *Faecalibaculum*, were also significantly decreased in the 10% NaCl group compared to 0.03% and 0.5% NaCl groups (Figure 3A). Overall, our results point to a reduction of probiotic bacteria to NaCl intake in a dose-dependent manner. On the other hand, *Akkermansia*, *Tuzzerella* and *Alistipes* were profoundly overrepresented in 10% NaCl compared to all other groups (Figure 3A and Figure S6). An enrichment of *Akkermansia* after high salt consumption was also observed in the study of Afroz et al. [39]. Lastly, to our knowledge, no study has detected any alterations in the representation of *Tuzzerella* after high salt intake (Figure 3A). 

Our results suggest that high salt intake leads to alterations in the gut microbiota marked by a depletion of lactic acid-producing bacteria and further overrepresentation of bacteria that were previously implicated on both inflammation and metabolic syndrome [40,41].

### 3.3. Functional Characterization of Bacterial Communities in Sodium-Depleted/Enriched Diets

Functional differences in community profiles were further investigated between the different groups by PICRUSt analysis. PICRUSt allows the prediction of metagenome functional content from 16s rRNA gene sequencing data. In our data, the proportion of sequences attributed to carbohydrate metabolism and amino acid metabolism changed significantly between the different diets (0.03% NaCl, 0.5% NaCl, 4% NaCl and 10% NaCl) (Figure 3B). Interestingly, PICRUSt predicted significant differences on the lipid metabolism between 4% NaCl and 10% NaCl-fed mice (Figure 3B). Further, a downregulation of pathways related to carbohydrate metabolism, such as starch degradation and pyruvate fermentation to propanoate, acetate and lactate, were observed in the HSD groups (4% NaCl and 10% NaCl) compared to the sodium deficient (0.03% NaCl) and control (0.5% NaCl) diets (Figure 3B,C).

Moreover, HSD seems to affect also the microbial pathways to NAD production, either from NAD+ biosynthesis reaction from aspartate or from NAD salvage from nicotinamide (Figure 3C). We also observed a decrease in the abundance of microbes involved in L-rhamnose degradation I (Figure 3C), which was found to contribute to 53% of the metabolites from healthy human fecal samples together with other pathways, such as NAD biosynthesis [42]. Interestingly, the PICRUSt2 analysis also predicted downregulation in genes involved in the tryptophan biosynthesis under HSD conditions (Figure 3C). Tryptophan metabolites are known to be involved in the enhancement of gut barrier functions and protect against inflammation caused by IBDs [43].

## 4. Discussion

High dietary salt intake has been shown to increase the risk to various diseases [13,44,45]. Previous studies in experimental animal models have shown that high-sodium diets containing 4% NaCl could profoundly modulate disease by altering immune responses and the gut microbiota composition [13,45]. However, the effects of other sodium ranges on the gut microbiome are unknown. We thus investigated the effects of different NaCl content in the food (0.03%, 0.5%, 4% and 10% NaCl) on the gut microbiome composition in conventionally housed C57BL/6 mice. Our results revealed that HSD (4% and 10% NaCl) led to distinct gut microbiota composition compared to sodium-depleted (0.03% NaCl) or control (0.5% NaCl) diets. NaCl content in the diet modulated gut microbiota composition particularly by decreasing the relative abundances of bacteria associated with immuno-competent SCFA production, such as *Bifidobacterium*, *Faecalibaculum*, *Blautia,* and *Lactobacillus* in a dose-dependent manner. 

These observations are in line with the studies from Wilck et al., Miranda et al. and Ferguson et al. [19,22,26], in which HSD-fed mice also revealed an underrepresentation of lactic acid producing bacteria. However, while our study and the work from Wilck and Miranda [19,26] detected a reduction of the *Lactobacillus* spp., the study from Ferguson uncovered different species being affected by HSD consumption [22]. This may be due to the fact that Ferguson et al. [22] used cecal samples rather than fecal samples to assess the microbiota composition of the gut. Interestingly, it has been previously shown that the cecal microbiome could greatly differ from that of feces [46].

In our study, the functional prediction profiles based on 16S rRNA amplicon sequencing showed a clear downregulation of pathways related to carbohydrate amino acid metabolism. It has been previously reported that some bacterial genera belonging to *Bifidobacterium*, *Faecalibaculum*, *Blautia* and *Lactobacillus* species are notoriously known for their ability to process non-digestible dietary fibers that include carbohydrate, such as starch, which cannot be absorbed in the small intestine of the host [47,48,49,50]. This leads to the release of SCFAs in the gut lumen, exerting tropic and immunomodulatory effects on host cells both regionally and systemically [51,52]. It is thus likely that the depletion of lactic acid-producing bacteria upon HSD, which are known to be involved in the metabolism of complex carbohydrates, contribute to the predicted downregulation of the carbohydrate metabolic pathways. 

Lower concentrations of fecal SCFAs or loss of bacterial SCFA-producers are usually correlated to dysbiosis, leading to intestinal inflammatory conditions, such as IBD [52,53,54,55]. Further, germfree (GF) mice, which are deficient in SCFAs in their gastrointestinal tract [56,57,58], have been shown to exhibit an exaggerated immune response in the DSS-induced colitis model [58]. Moreover, recent studies successfully proved that SCFA producers participate in the establishment of a healthy gut-brain axis [59,60,61]. SCFAs have been proven to mitigate experimental autoimmune encephalomyelitis (EAE) in mice by promoting the differentiation of Treg cells over Th1/Th17 cells [62]. Our results are in agreement with previous studies exploring the impact of salt on the gut microbiome [19,26,39], in which SCFA-producing bacteria, such as *Lactobacillus* were found to be significantly decreased after HSD. In addition to *Lactobacillus*, we also identified other bacterial taxa belonging to the *Bifidobacterium* and *Faecalibaculum*. Neither of these taxa had been previously identified as being affected by HSD. 

Furthermore, we identified a significant increase in the abundance of genus *Akkermansia* in HSD compared to control-fed mice. Similar results were reported by Afroz et al. [39], in which a significant enrichment of *Akkermansia* in mice was observed in the 8% HSD compared to the control-fed mice. Interestingly, species such as *Akkermansia muciniphila*, have previously been reported to have proinflammatory functions in vitro [63] and also found to be enriched in the gut of MS patients compared to controls [63,64,65]. Finally, our results revealed that the member of genus *Alistipes,* which are highly relevant in dysbiosis and chronic intestinal inflammation, were overrepresented in HSD fed mice [66]. This result is contradicting previous findings of Wang et al. [14], who detected low abundances of *Alistepes* under HSD. This could potentially be due to the fact that Wang et al. used different NaCl ranges in the diet (0.25 vs. 3.15% NaCl). Additionally, they fed the mice for 8 weeks, which represents a long period of time in comparison to our study.

In conclusion, our study represents a comprehensive characterization of the impact of different sodium contents on the gut microbiome in mice. Our findings show that HSD cause disturbances in the ecological balance of the gastrointestinal microflora, primarily through depletion of lactic acid-producing bacteria in a dose-dependent manner. These findings may thus have important implications for salt-sensitive inflammatory diseases uncovering salt-dependent immunomodulatory microbial signatures.

## Data Availability

The data sets used and/or analyzed during the current study are available from the corresponding author upon reasonable request.

## Supplementary Materials

The following supporting information can be downloaded at: https://www.mdpi.com/article/10.3390/nu14061171/s1, Figure S1: Rarefaction curves indicating the observed number of operational taxonomic units (OTUs) within a sample. Rarefaction curves of OTUs clustered and saturated at different levels across exposure groups and indicate the intra-sample diversity. Figure S2: A Venn diagram depicting the shared and unique bacterial OTUs of the gut microbiome among mice fed on sodium-deficient 0.03% NaCl, 0.5% NaCl (control), high salt 4% NaCl (HSD) and high salt 10% NaCl (HSD). Figure S3: Alpha diversity analysis of fecal microbiota, a boxplot depicting the Shannon diversity, InvSimpson and Simpson’s diversity index, which is a measure for the diversity of the gut microbiome. Figure S4: PCoA of unweighted and weighted UniFrac distances (upper and middle). PCoA results calculated with unweighted UniFrac distances depicting the beta diversity of gut microbiota among mice fed on sodium-deficient 0.03% NaCl, 0.5% NaCl (control), high salt 4% NaCl (HSD) and high salt 10% NaCl (HSD). Conditional correspondence analysis constrained by %NaCl content (bottom), the analysis of similarity was performed by the anosim function from “vegan” package of R, *p* value = 0.001. Figure S5: Bacterial families differentially represented in feces of mice fed on sodium-deficient 0.03% NaCl, 0.5% NaCl (control), high salt 4% NaCl (HSD) and high salt 10% NaCl (HSD). Figure S6: Bacterial genera differentially represented in feces of mice fed on sodium-deficient 0.03% NaCl, 0.5% NaCl (control), high salt 4% NaCl (HSD) and high salt 10% NaCl (HSD).
