# Supplementary material for: High-Salt Diet Induces Depletion of Lactic Acid-Producing Bacteria in Murine Gut
Source: Nutrients. 2022 Mar 10;14(6):1171. doi: 10.3390/nu14061171 (PMC8950745; doi:10.3390/nu14061171)
Supplement: Supplementary file 1 [file nutrients-14-01171-s001.zip › nutrients-1600112-supplementary-done.pdf]

nutrients

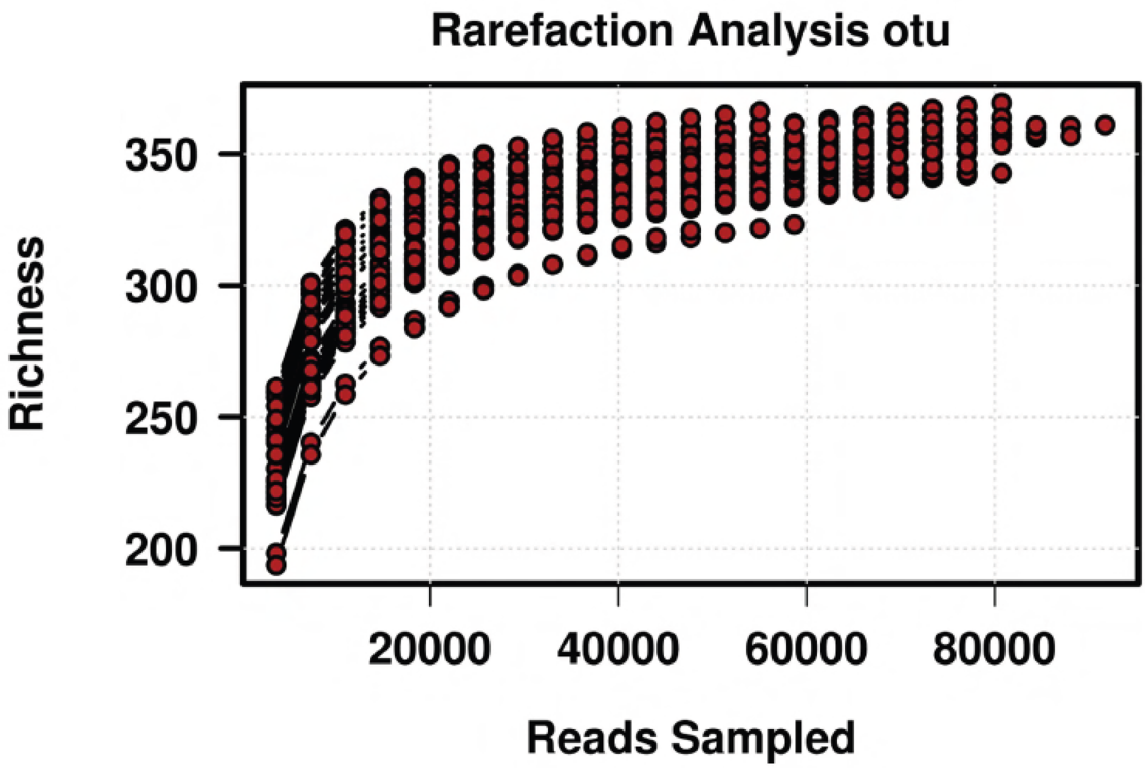


**Figure S1.** Rarefaction curves indicating the observed number of operational taxonomic units (OTUs) within a sample. Rarefaction curves of OTUs clustered and saturated at different levels across exposure groups and indicate the intra-sample diversity.

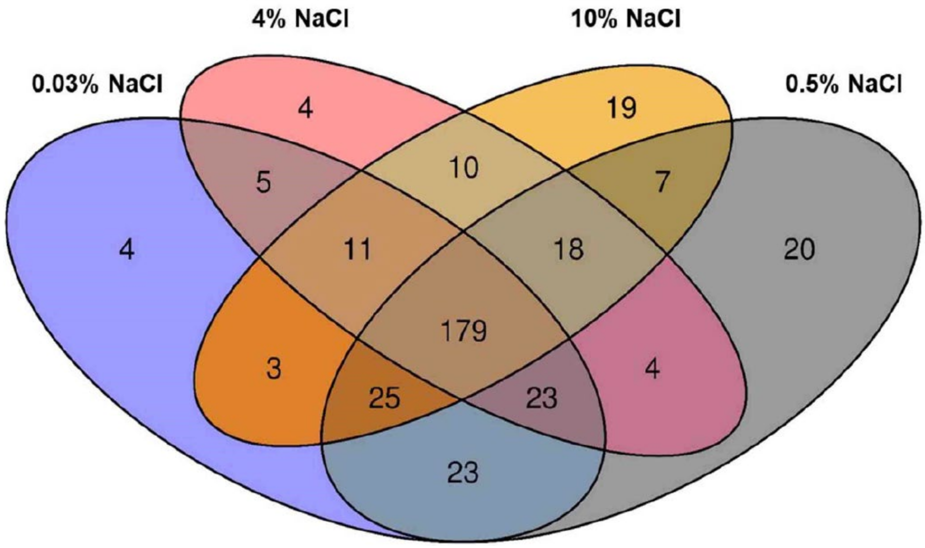

| Bacterial OTUs | Type | Food content |
|---|---|---|
| p__Firmicutes__g__Blautia_otu282 | unique | 0.03% NaCl |
| p__Firmicutes__f__Lachnospiraceae_otu314 | unique | 0.03% NaCl |
| p__Firmicutes__f__Lachnospiraceae_otu274 | unique | 0.03% NaCl |
| p__Actinobacteriota__g__Bifidobacterium_otu183 | unique | 0.03% NaCl |
| p__Firmicutes__g__uncultured__s__unidentified_otu395 | unique | 0.5% NaCl |
| p__Firmicutes__g__uncultured__s__unidentified_otu260 | unique | 0.5% NaCl |
| p__Firmicutes__g__uncultured__s__Clostridiales_bacterium_otu298 | unique | 0.5% NaCl |
| p__Firmicutes__g__Oscillibacter__s__Clostridium_leptum_otu766 | unique | 0.5% NaCl |
| p__Firmicutes__g__Oscillibacter__s__Clostridiales_bacterium_otu247 | unique | 0.5% NaCl |
| p__Firmicutes__g__Clostridia_UCG014_otu71 | unique | 0.5% NaCl |
| p__Firmicutes__g__Blautia_otu495 | unique | 0.5% NaCl |
| p__Firmicutes__g__Blautia_otu479 | unique | 0.5% NaCl |
| p__Firmicutes__g__Anaeroplasma__s__uncultured_Anaeroplasma_otu369 | unique | 0.5% NaCl |
| p__Firmicutes__g__Acetatifactor__s__Acetatifactor_muris_otu649 | unique | 0.5% NaCl |
| p__Firmicutes__f__Peptostreptococcaceae_otu663 | unique | 0.5% NaCl |
| p__Firmicutes__f__Lachnospiraceae_otu598 | unique | 0.5% NaCl |
| p__Firmicutes__f__Lachnospiraceae_otu370 | unique | 0.5% NaCl |
| p__Firmicutes__f__Lachnospiraceae_otu354 | unique | 0.5% NaCl |
| p__Firmicutes__f__Lachnospiraceae_otu336 | unique | 0.5% NaCl |
| p__Firmicutes__f__Lachnospiraceae_otu276 | unique | 0.5% NaCl |
| p__Firmicutes__f__Lachnospiraceae_otu272 | unique | 0.5% NaCl |
| p__Firmicutes__f__Lachnospiraceae_otu193 | unique | 0.5% NaCl |
| p__Bacteroidota__g__Muribaculaceae__s__uncultured_bacterium_otu208 | unique | 0.5% NaCl |
| p__Actinobacteriota__g__Bifidobacterium_otu340 | unique | 0.5% NaCl |
| p__Firmicutes__g__Clostridia_UCG014__s__uncultured_Escherichia_otu236 | unique | 4% NaCl |
| p__Firmicutes__g__Blautia__s__uncultured_Clostridiales_otu600 | unique | 4% NaCl |
| p__Firmicutes__g__Blautia__s__uncultured_Clostridiales_otu580 | unique | 4% NaCl |
| p__Firmicutes__g__Blautia__s__uncultured_Clostridiales_otu519 | unique | 4% NaCl |
| p__Proteobacteria__o__Enterobacterales_otu92 | unique | 10% NaCl |
| p__Proteobacteria__o__Enterobacterales_otu62 | unique | 10% NaCl |
| p__Proteobacteria__o__Enterobacterales_otu301 | unique | 10% NaCl |
| p__Firmicutes__g__uncultured__s__unidentified_otu261 | unique | 10% NaCl |
| p__Firmicutes__g__Roseburia_otu248 | unique | 10% NaCl |
| p__Firmicutes__g__Roseburia_otu245 | unique | 10% NaCl |
| p__Firmicutes__g__Lachnospiraceae_UCG006__s__Clostridium_sp._otu325 | unique | 10% NaCl |
| p__Firmicutes__g__Lachnoclostridium_otu306 | unique | 10% NaCl |
| p__Firmicutes__g__Incertae_Sedis_otu321 | unique | 10% NaCl |
| p__Firmicutes__g__Colidextribacter_otu400 | unique | 10% NaCl |
| p__Firmicutes__g__Clostridia_UCG014__s__uncultured_Escherichia_otu161 | unique | 10% NaCl |
| p__Firmicutes__g__Acetatifactor__s__Acetatifactor_muris_otu293 | unique | 10% NaCl |
| p__Firmicutes__f__Oscillospiraceae_otu327 | unique | 10% NaCl |
| p__Firmicutes__f__Oscillospiraceae_otu317 | unique | 10% NaCl |
| p__Firmicutes__f__Oscillospiraceae_otu308 | unique | 10% NaCl |
| p__Firmicutes__f__Oscillospiraceae_otu228 | unique | 10% NaCl |
| p__Firmicutes__f__Lachnospiraceae_otu407 | unique | 10% NaCl |
| p__Bacteroidota__g__Muribaculum__s__Muribaculum_intestinale_otu200 | unique | 10% NaCl |
| p__Bacteroidota__g__Muribaculaceae__s__uncultured_bacterium_otu190 | unique | 10% NaCl |

**Figure S2.** A Venn diagram depicting the shared and unique bacterial OTUs of the gut microbiome among mice fed on sodium-deficient 0.03% NaCl, 0.5% NaCl (control), high salt 4% NaCl (HSD) and high salt 10% NaCl (HSD).

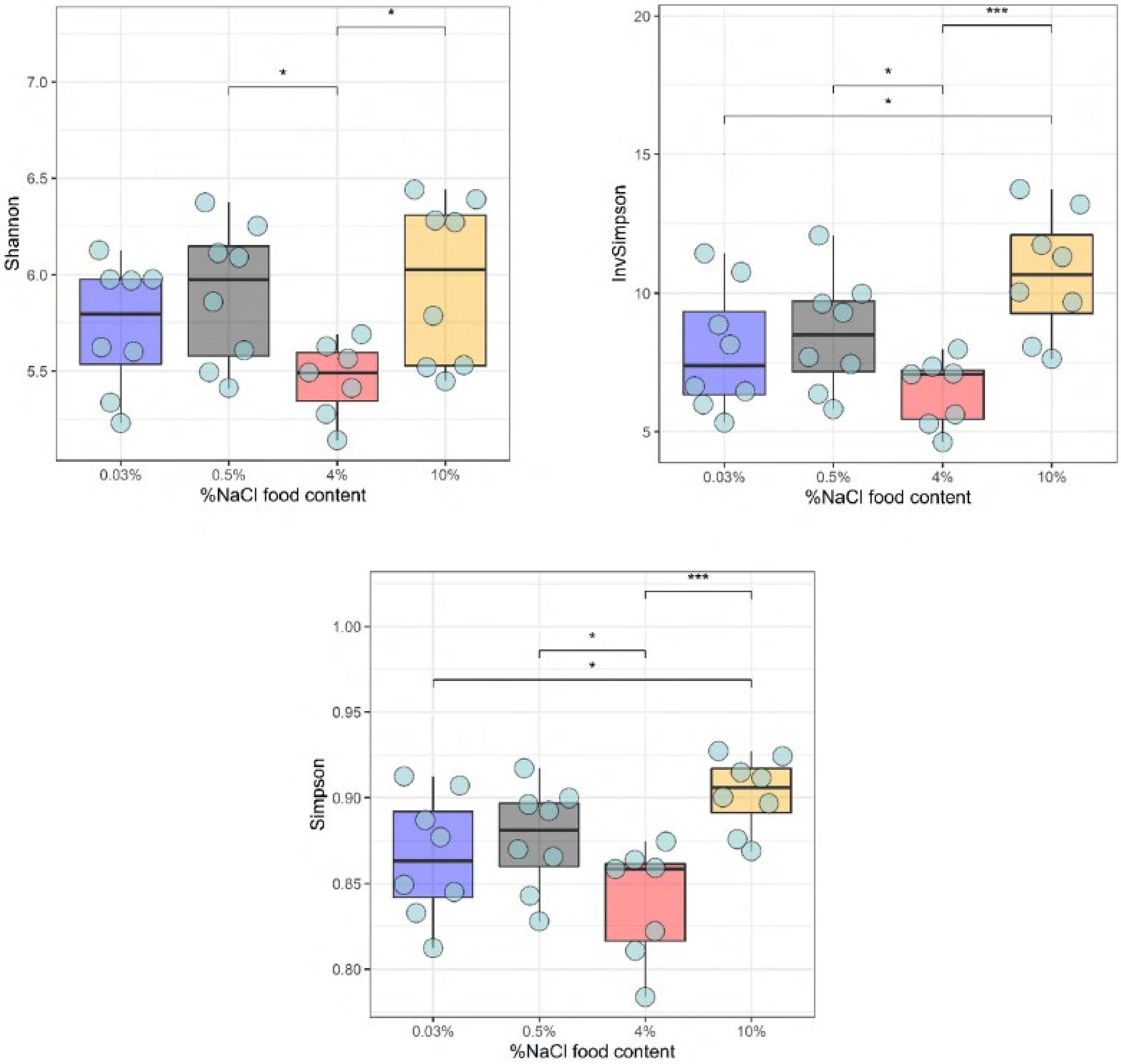


**Figure S3.** Alpha diversity analysis of fecal microbiota, a boxplot depicting the Shannon diversity, InvSimpson and Simpson's diversity index, which is a measure for the diversity of the gut microbiome. * p ≤ 0.05; **; *** p ≤ 0.001.

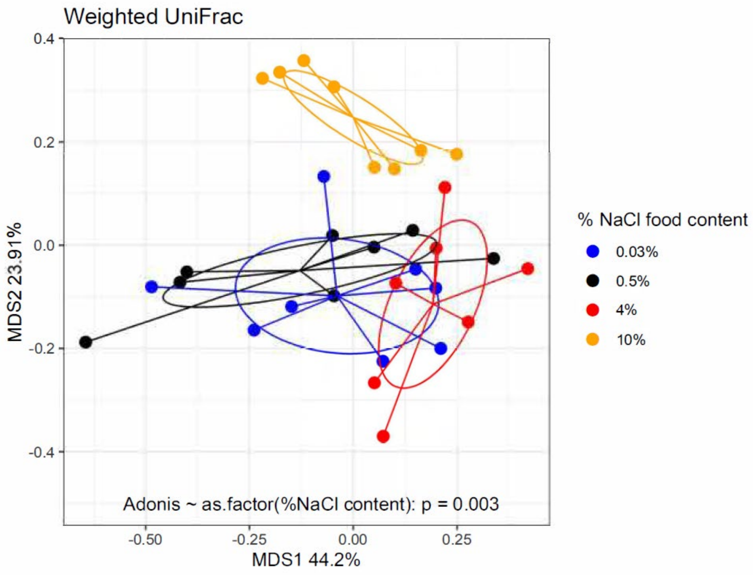

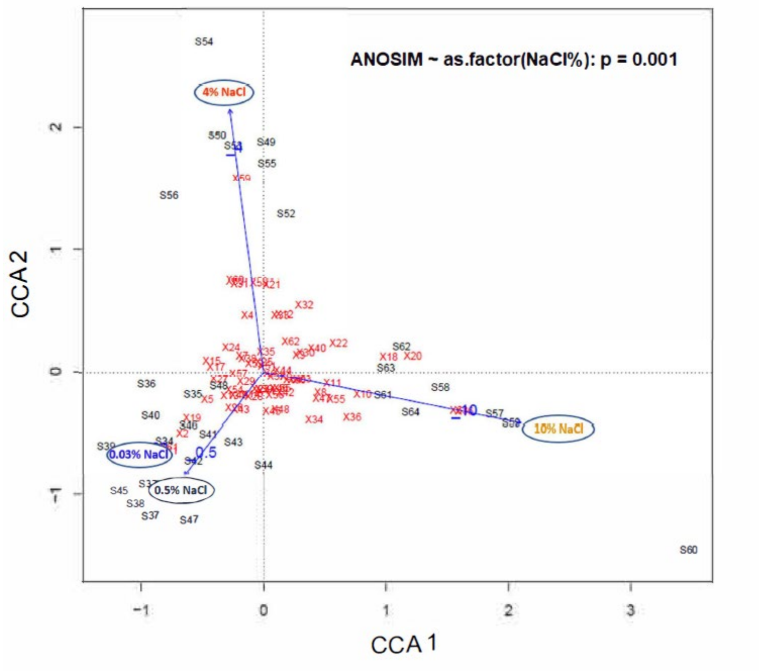


**Figure S4.** PCoA of unweighted and weighted UniFrac distances (upper and middle). PCoA results calculated with unweighted UniFrac distances depicting the beta diversity of gut microbiota among mice fed on sodium-deficient 0.03% NaCl, 0.5% NaCl (control), high salt 4% NaCl (HSD) and high salt 10% NaCl (HSD). Conditional correspondence analysis constrained by %NaCl content (bottom), the analysis of similarity was performed by the anosim function from "vegan" package of R, *p* value = 0.001.

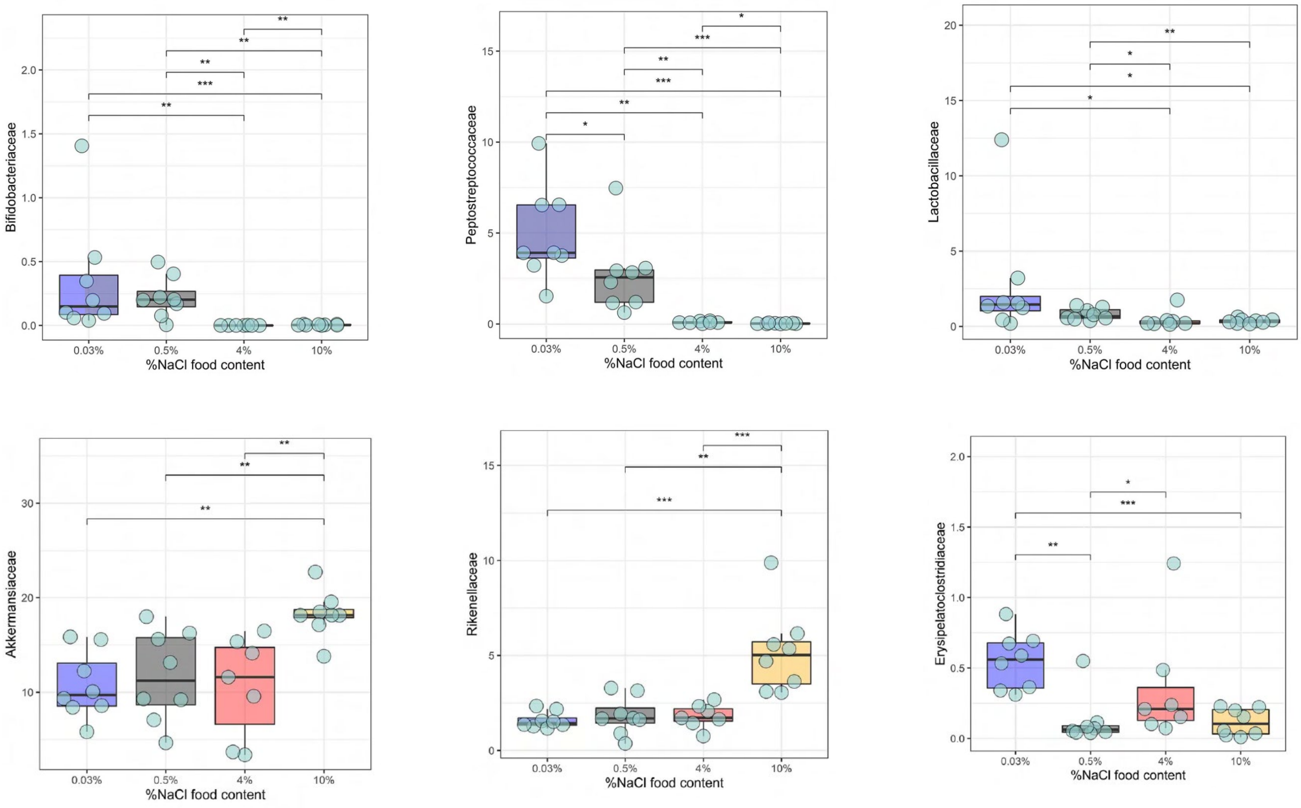

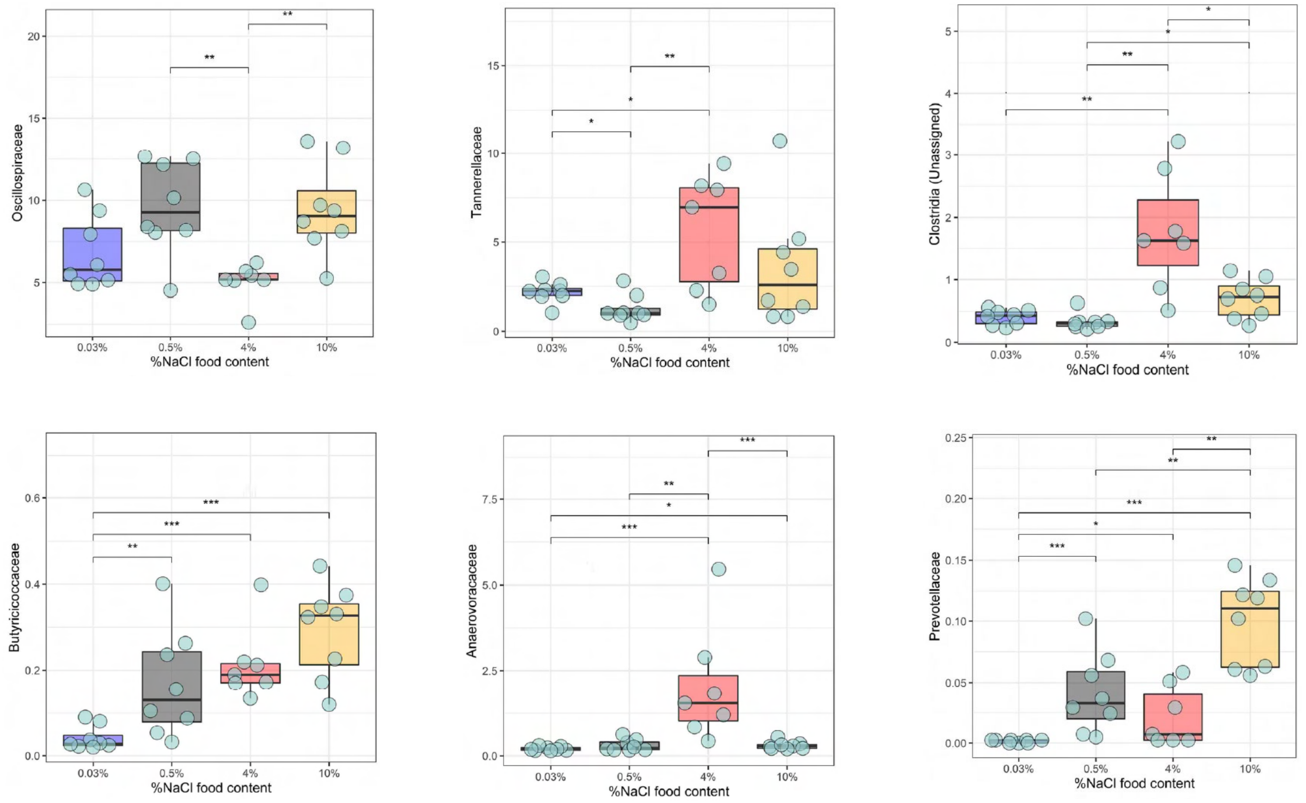


**Figure S5.** Bacterial families differentially represented in feces of mice fed on sodium-deficient 0.03% NaCl, 0.5% NaCl (control), high salt 4% NaCl (HSD) and high salt 10% NaCl (HSD). * p ≤ 0.05; ** p ≤ 0.01; *** p ≤ 0.001.

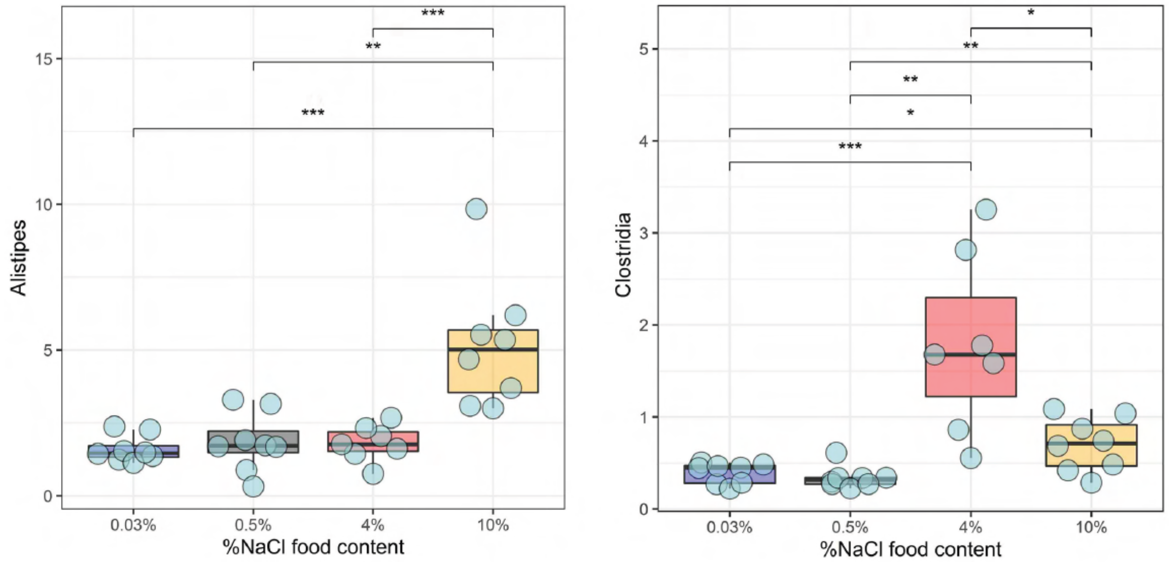


**Figure S6.** Bacterial genera differentially represented in feces of mice fed on sodium-deficient 0.03% NaCl, 0.5% NaCl (control), high salt 4% NaCl (HSD) and high salt 10% NaCl (HSD). ). * p ≤ 0.05; ** p ≤ 0.01; *** p ≤ 0.001.
